# Multi-Kernel Learning with Dartel Improves Combined MRI-PET Classification of Alzheimer’s Disease in AIBL Data: Group and Individual Analyses

**DOI:** 10.3389/fnhum.2017.00380

**Published:** 2017-07-25

**Authors:** Vahab Youssofzadeh, Bernadette McGuinness, Liam P. Maguire, KongFatt Wong-Lin

**Affiliations:** ^1^Computational Neuroscience Research Team, Intelligent Systems Research Centre, School of Computing and Intelligent Systems, Faculty of Computing and Engineering, Ulster University Londonderry, United Kingdom; ^2^Division of Neurology, Cincinnati Children’s Hospital Medical Center Cincinnati, OH, United States; ^3^Institute of Clinical Science B, Centre for Public Health, Queen’s University Belfast Belfast, United Kingdom

**Keywords:** Alzheimer’s disease, classification, machine learning, multi-kernel learning, prediction, Australian imaging, biomarkers, lifestyle AIBL

## Abstract

Magnetic resonance imaging (MRI) and positron emission tomography (PET) are neuroimaging modalities typically used for evaluating brain changes in Alzheimer’s disease (AD). Due to their complementary nature, their combination can provide more accurate AD diagnosis or prognosis. In this work, we apply a multi-modal imaging machine-learning framework to enhance AD classification and prediction of diagnosis of subject-matched gray matter MRI and Pittsburgh compound B (PiB)-PET data related to 58 AD, 108 mild cognitive impairment (MCI) and 120 healthy elderly (HE) subjects from the Australian imaging, biomarkers and lifestyle (AIBL) dataset. Specifically, we combined a Dartel algorithm to enhance anatomical registration with multi-kernel learning (MKL) technique, yielding an average of >95% accuracy for three binary classification problems: AD-vs.-HE, MCI-vs.-HE and AD-vs.-MCI, a considerable improvement from individual modality approach. Consistent with *t*-contrasts, the MKL weight maps revealed known brain regions associated with AD, i.e., (para)hippocampus, posterior cingulate cortex and bilateral temporal gyrus. Importantly, MKL regression analysis provided excellent predictions of diagnosis of individuals by *r*^2^ = 0.86. In addition, we found significant correlations between the MKL classification and delayed memory recall scores with *r*^2^ = 0.62 (*p* < 0.01). Interestingly, outliers in the regression model for diagnosis were mainly converter samples with a higher likelihood of converting to the inclined diagnostic category. Overall, our work demonstrates the successful application of MKL with Dartel on combined neuromarkers from different neuroimaging modalities in the AIBL data. This lends further support in favor of machine learning approach in improving the diagnosis and risk prediction of AD.

## Introduction

Magnetic resonance imaging (MRI) and positron emission tomography (PET) are two imaging data modalities that are routinely used for evaluating changes in the brain associated with Alzheimer’s disease (AD) (Ewers et al., 2011; Bateman et al., 2012). Biomarkers from these neuroimaging data are crucial for identifying early symptoms of AD pathology. For example, structural atrophy measured by MRI, and glucose metabolism or amyloid-β deposition measured by PET scans can be detected almost 15–20 years before the expected symptom onset as compared to 5 years in advance by clinical tests (Bateman et al., 2012; Weiner et al., 2013). In addition, biomarkers from neuroimaging data are more sensitive and reliable measures of AD progression than the cognitive and clinical assessments (Ye et al., 2008).

Previous MR-based studies have found brain atrophy in subcortical regions such as the hippocampal pathway (entorhinal cortex, hippocampus, and posterior cingulate cortex; Frisoni et al., 2010; Bateman et al., 2012) and cortical thickness reduction at vulnerable regions (Desikan et al., 2009; Dickerson et al., 2009) at the earliest stages of the disease. Moreover, longitudinal PET studies with [^18^F]-fluorodeoxyglucose (FDG) tracer have reported reduced cerebral metabolic rate of glucose (hypometabolism) in bilateral parietotemporal, frontal and posterior cingulate cortices in AD and mild cognitive impairment (MCI) participants with respect to healthy elderly (HE) participants (Mosconi et al., 2005; Nordberg et al., 2010) or in baseline-vs.-follow-up whole-group study (Apostolova et al., 2010). Other PET studies with [^11^C]-Pittsburgh compound B (PiB) tracer have found an increase of cortical PiB retention in areas known to significantly accumulate amyloid-beta [Aβ] deposits in AD and MCI subjects with respect to HE (Nordberg et al., 2010; Cohen and Klunk, 2014). However, in most cases, one biomarker is not sufficient for an accurate diagnosis or prognosis of the disease because each modality reveals information about different aspects of the underlying pathology (Hinrichs et al., 2011).

Recently, multi-modal imaging has gained popularity by integrating complementary AD characterization, and hence obtaining a more reliable AD biomarker (Cuingnet et al., 2011; Dukart et al., 2011; Zhang et al., 2011). In particular, multi-kernel learning (MKL) is a useful machine learning technique to enhance interpretability and classification accuracy of multi-modal imaging (Wang et al., 2008; Ye et al., 2008; Hinrichs et al., 2009; Zhang et al., 2011; Dai et al., 2012; Segovia et al., 2014). Specifically, the MKL forms an optimal kernel from a linear combination of kernels/features (Sonnenburg et al., 2006; Gönen and Alpaydin, 2011). Importantly, MKL can be easily embedded in a support vector machine (SVM) for high-dimensional pattern classification/recognition (Gönen and Alpaydin, 2011). The SVM relies on the assumption that (two) classes are separable by linear decision boundary (separating hyperplane) in a feature space (transformed features via a non-linear transformation function; Cortes and Vapnik, 1995). MKL simultaneously optimizes weights under a gradient descent algorithm and maximizes the margin in SVM (Rakotomamonjy et al., 2008). Previous works have utilized the MKL method on AD neuroimaging initiative (ADNI) data and reported substantial improvement in classification performance, an accuracy rate of above 90% (Hinrichs et al., 2009, 2011; Zhang et al., 2011; Zhang and Shen, 2012). Tested on ADNI dataset, MKL can outperform SVM by 3%–4% and enable early AD diagnosis e.g., separating converting vs. non-converting MCI (Hinrichs et al., 2011).

Dartel is a suite of tools to enhance inter-subject registration or spatial normalization of anatomical scans, allowing for less smoothing and improving MRI-PET coregistration (Ashburner, 2007). This leads to improved anatomical precision (Bergouignan et al., 2009; Klein et al., 2009; Yassa and Stark, 2009). In addition, improvements of AD classification of the ADNI dataset have been reported using Dartel registration as compared to unified segmentation (Cuingnet et al., 2011). However, previous work has only applied to structural neuroimaging data.

**Figure 1:**
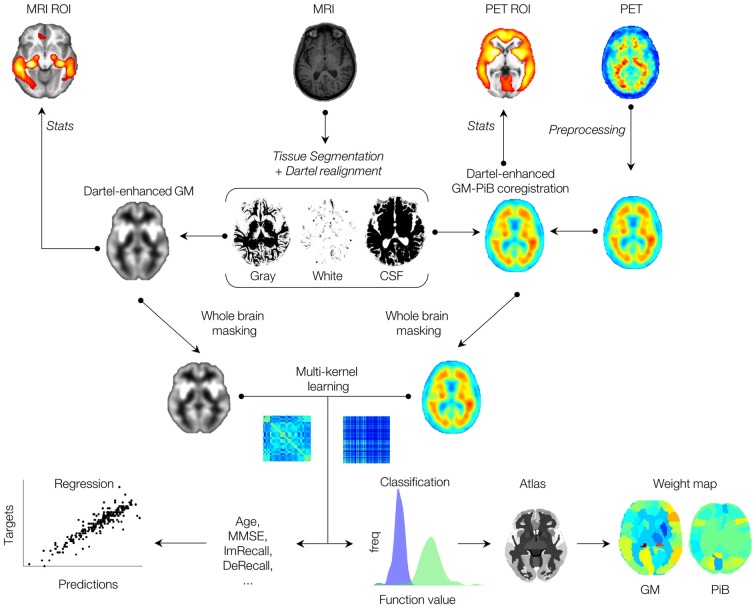
Schematic overview of the proposed multimodal (imaging) machine-learning framework. See text for detailed descriptions.

In the present work, we use a multimodal machine-learning framework, utilizing both MKL and Dartel techniques, to enhance multimodal classification accuracy of imaging scans from AIBL dataset. A flowchart of the framework is presented in Figure 1, where both MRI and PET imaging modalities after anatomical coregistration enhancement by the Dartel algorithm are jointly combined via a multi-kernel learning process. Contributions of ROIs are derived by whole-brain MKL weights, and results are compared with the *t*-contrasts of conventional general linear model (GLM) analysis. In addition, expected ranking values are computed for each data modality, indicating variability (stability) ranking of regions across the folds. Using the Australian imaging, biomarkers, and lifestyle (AIBL) data, our results showed considerable improvement of combined MRI-PET classification accuracy over single modal approach, and correlated strongly with the scores of a commonly used psychological test, the delayed memory recall test. Importantly, our results showed high diagnostic accuracy for individual samples and can potentially predict the likelihood of individual’s stability or conversion to another AD category.

## Materials and Methods

### Data Characteristics

As shown in Table 1, for AD with respect to MCI and HE participants, the average Mini-Mental State Examination (MMSE) score was the lowest (20 ± 2.1), while the average clinical dementia rating (CDR) scores (0.85 ± 0.4) and average delayed memory recall (DeRecall) scores were the highest (1.28 ± 2.0). Expectedly, MCI participants showed lower average MMSE and DeRecall scores and higher average CDR scores with respect to HE subjects. These graded scores fall within the standard range of diagnosis, and hence confirmed the validity of the diagnosis, which will be used as targets in the supervised machine learning process.

**Table 1:** Demographic and neuropsychological characteristics of the study population from 58 AD, 108 MCI and 120 HE samples.

Total scans: 286	AD		MCI		HE	
	(*n* = 58; 23 F/35 M)		(*n* = 108; 47 F/61 M)		(*n* = 120; 55 F/65 M)	
	Mean ± SD	Range	Mean ± SD	Range	Mean ± SD	Range
Age	74.3 ± 4.1	55–93	75.1 ± 5.3	60–96	72.9 ± 5.1	55–93
MMSE	20 ± 2.1	2–29	27 ± 2.3	20–30	28.7 ± 2.2	24–30
CDR	0.85 ± 0.4	0.5–3	0.4 ± 0.2	0–0.5	0.02 ± 0.23	0–0.5
DeRecall	1.28 ± 2.08	0–8	4.83 ± 3.93	0–17	11.26 ± 3.95	0–22

*The numbers refer to subjects. AD, Alzheimer’s Disease, MCI, Mild Cognitive Impairment, HE, Healthy Elderly, MMSE, Mini-Mental State Examination, CDR, Clinical Dementia Ratio; SD, standard deviation*.

### MRI and PET Data

The imaging data from the AIBL flagship study of ageing (Ellis et al., 2009) dataset was used in this study. Data was collected by the AIBL study group. AIBL study methodology has been reported previously (Ellis et al., 2009; Albrecht et al., 2015; Gupta et al., 2015). Demographic characteristics of the studied population of the AIBL data are shown in Table 1. To test the multi-modal machine-learning framework, subject-matched MRI and Pittsburgh compound B-positron emission tomography (PiB-PET) imaging data clinically diagnosed with AD (= 58), MCI (= 108), and HE (= 120) were analyzed. Among them, there were four MCI converters (2 MCI-to-AD and 2 MCI-to-HE), two AD converters (2 AD-to-MCI) and four HE converters (HE-to-MCI), from baseline (BL) to a later time (month 18 or later), which were used to partially evaluate the regression models.

**Figure 2:**
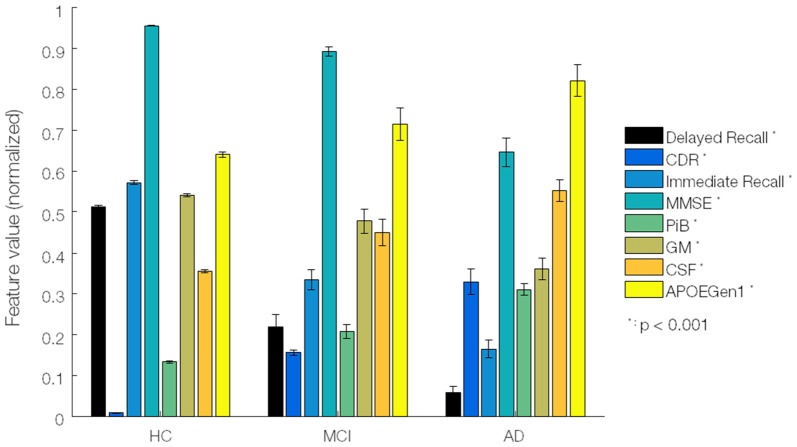
Significant data features ranked by ANOVA test. A total of 8 out of 33 features were identified by ANOVA test from 58 Alzheimer’s disease (AD), 108 mild cognitive impairment (MCI) and 120 healthy elderly (HE) samples. Features/biomarkers were ranked based on their *p*-values. The biomarkers include clinical dementia ratio (CDR), Mini-Mental State Exam (MMSE), delayed memory recall (DeRecall), immediate memory recall (Immediate Recall), gray matter (GM), average intensity of PiB, cerebrospinal fluid (CSF), and Apolipoprotein E (ApoE) genotype 1. Error bars represent a standard error.

Although our focus was on imaging data, the relationships with non-imaging data were also investigated. In particular, delayed memory recall (DeRecall) score was tested by the MKL regression model to check how well such non-categorical scores can correlate with the imaging features/kernels. As presented in Figure 2, by means of a one-way ANOVA test, we assessed 33 (29 non-imaging + 4 imaging) features to identify the most distinctive markers among three diagnostic groups of samples. The non-imaging features considered were gender, age, neuropsychology test scores (MMSE, CDR and delayed/immediate recall memory tests), blood test analysis (Apolipoprotein E or ApoE genotypes and hormones including thyroid stim, vitamin B12, red blood cells, nucleated red blood cells, platelets, hemoglobin, mean corpuscular hemoglobin (MCH), MCH concentration, urea nitrogen, serum glucose, cholesterol and creatinine), medical history (psychiatric, neurological, cardiovascular, hepatic, musculoskeletal, endocrine-metabolic, gastrointestinal, renal-genitourinary, smoking, malignancy), laboratory data (thyroid hormone, vitamin B12, red blood cell count, white blood cell count, platelets, hemoglobin, MCH, MCH concentration, urea nitrogen, serum glucose, cholesterol, creatinine). The imaging features were the mean intracranial volume of gray matter (GM), white matter (WM) and cerebrospinal fluid (CSF) brain tissues, and average voxel intensity of PIB-PET scans. The features were normalized in [0, 1] (each divided by its maximum) before applying the ANOVA test to select and rank the top features in terms of significance with respect to AD progression.

### Processing of MRI and PET Scans

A total number of 286 (58 AD, 108 MCI and 120 HE) MRI scans were evaluated using a Dartel-enhanced voxel-based morphometry (VBM) analysis, as interfaced in SPM12 (Wellcome Trust Centre for Neuroimaging, London)^1^. Scans were acquired with a T1-weighted magnetization-prepared rapid gradient echo (MPRAGE) sequence using 1.5T scanners (repetition time/echo time/inversion time = 2300/3.04/900 ms and flip angle = 9°) in DICOM format. Steps to process the MRI scans were as follows: (1) origins of scans were set to the anterior commissure (AC) brain area; (2) MR scans were segmented into GM, WM and CSF brain tissues. Accordingly, “c_1_”, “c_2_” and “c_3_” NIFTI files were generated. In addition, Dartel imported versions of tissue class images for GM and WM i.e., “rc_1_” and “rc_2_” were obtained; (3) the “rc_1_” and “rc_2_” images were used to create a template from the mean of all scans/subjects. In addition, they were used to generate flow fields that contain deformation details of scans; (4) GMs (“c_1_”) were normalized into a Montreal Neurological Institute (MNI) space using the Dartel template (“Template_6”) and flow fields (“u_rc_1_”). To compensate for any residual due to inter-subject variability and, to increase the signal-to-noise ratio, spatially smoothing was applied to GMs (“c_1_”) images using a full-width-at-half-maximum (FWHM) Gaussian kernel with a common resolution of 8 mm. The resulted “smwc_1_” scans were then used for the unpaired two-sample *t*-test.

An equal number of PiB-PET scans were separately preprocessed. Scans were aligned, co-registered to the Dartel-enhanced average of GM scans (maximization of mutual information), normalized into MNI space and smoothed with FWHM = 8 mm. The resulted scans (image matrix dimension 121 × 145 × 121 with 1.5 × 1.5 × 1.5 mm spacing) were used for the unpaired two-sample *t*-test, and later for the multi-modal machine-learning process.

An unpaired two-sample *t*-test was separately applied to structural MR and PiB-PET scans to look for differences between three groups of subjects. A threshold of 0.05 with a family-wise error correction for multiple comparisons at the voxel-level was employed. Comparisons for MRI and PiB-PET were made based on the following contrasts: subtraction of AD from HE (AD-vs.-HE), MCI-vs.-HE and AD-vs.-MCI. In regards to *t*-statistics, we looked at regions of interests (ROIs) with maximum *t*-values. For MRI scans, we expected to identify significant differences in (para)hippocampus, temporal gyrus and posterior cingulate cortex regions (Frisoni et al., 2010; Bateman et al., 2012). For PiB-PET scans, we expected to see significant differences at bilateral parietotemporal, frontal and posterior cingulate cortices (Langbaum et al., 2009; Villain et al., 2012).

### Multimodal Classification and Regression Analyses

Kernels per modality were built from subject matched whole-brain GM and PiB image scans (of 58 AD, 108 MCI, and 120 HE subjects), as implemented in PRoNTo.v2 (Schrouff et al., 2013b). Kernels were simply the pairwise similarity measures (dot product) between scan pairs per region (LaConte et al., 2005). An anatomical automatic labeling (AAL) atlas consisting of 90 ROIs was used to parcellate the regions (Tzourio-Mazoyer et al., 2002). An optimized MKL technique called “simpleMKL” or sMKL was used to combine multiple kernels of GM and PiB scans. The sMKL is an iterative method that results in a smooth and convex optimization problem (Rakotomamonjy et al., 2008). It works based on a weighted *ℓ*_2_-norm regularization and sparsity (of linear combinations of the kernel) controlled by *ℓ*_1_-norm constrains. This makes it superior to other similar algorithms in terms of convergence and efficiency. A whole-brain binary mask (provided by GLM analysis of GMs) was applied to both data modalities. The first-level masking discards all uninteresting features such as voxels outside the brain.

A nested cross-validation (CV) with hyperparameter optimization was used for assessment of the generalization error (Marquand et al., 2010). For the inner loop, 10-fold CV on subjects-per-group-out and for the outer loop, leave-one-subject-out techniques were used. The 10-fold CV was chosen for the inner loop since it had fewer folds and reduced the computational time. The sMKL employs a binary SVM for classification. We used a soft-margin hyper-parameter optimization with the best configuration among *C* = 0.1, 1, 10 and 100. All the C values were tested using a 10-fold cross validation (inner folds), then the best C value was used for the outer loop.

To have an unbiased classification, kernels were mean-centered and normalized) $f_{i}=(f_{i}-{\bar{f}}_{i})/\sigma_{i},$ *f_i_* and *σ_i_* are the mean and standard deviation of *i*-th sample, respectively). This is due to fact that kernels can be computed from samples with a different number of features (i.e., regions with different numbers of voxels). A balanced accuracy (BA) = 0.5 × (CA1 + CA2), where CA1 and CA1 were accuracies of class 1 and 2 was used to report the overall performance (Schrouff et al., 2013c). Due to inherent imbalance data, we also reported class accuracies. The AAL atlas was used to construct the sMKL weight maps. The weight maps were the spatial representation of the decision function that defined the level of ROIs contributions to the classification process. In addition to percentage contribution, expected ranking values were computed for each data modality, indicating variability (stability) ranking of the regions across folds (Schrouff et al., 2013a).

Following the classification, an sMKL regression analysis was applied to multimodal data to make predictions about age, diagnosis and psychological (DeRecall) scores of subjects. The regression accuracies were compared with individual data, which were modeled by kernel ridge regression (KRR) method (Shawe-Taylor and Cristianini, 2004). Similar to classification process, a nested cross validation technique was used to report the generalization error. Kernels were mean-centered and normalized. To assess the goodness-of-fit of the regression models the coefficient of determination (*r*^2^) based on Pearson’s correlation was computed. Confidence intervals (*p*-values) generated by non-parametric permutation testing with 5000 randomizations were used to assure low variability in the outputs of classification/regression models.

## Results

Based on the ANOVA test, we identified eight features which were significantly linked to AD progression. Specifically, the ranking in terms of the highest significance to AD progression was CDR, DeRecall, immediate memory recall, MMSE, PiB-PET, GM-MRI, CSF-MRI and ApoE genotypes (Figure 2). We henceforth based our study on these relatively more significant features. In particular, with regard to imaging data, we considered GM-MRI and PiB-PET data modalities. By default, as a known key risk factor for AD, we also included age in our (regression) analyses.

### GLM Analysis: Group Statistical Analysis of MRI and PET Scans

**Figure 3:**
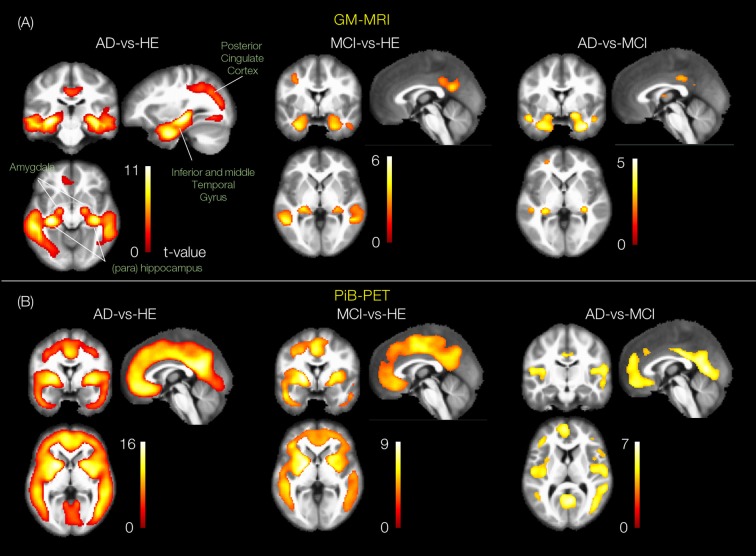
Group statistical differences of the group pairs for GM-MRI and Pittsburgh compound B-positron emission tomography (PiB-PET) volumes. The rendered *t*-contrast maps obtained from unpaired two sample *t*-test factorial analysis of three group pairs, AD-vs.-HE, MCI-vs.-HE and AD-vs.-MCI for **(A)** GM and **(B)** PiB scans.

Group *t*-statistics of GMs in AD-vs.-HE contrast revealed significant changes in bilateral (left and right) subcortical regions: hippocampus (*t* = 11.0 and 8.7), parahippocampus (*t* = 7.0 and 9.5), fusiform gyrus (*t* = 10.8 and 10.34) as well as in bilateral cortical regions: middle temporal gyrus (*t* = 8.1 and 7.8), inferior temporal gyrus (*t* = 7.1 and 6.7) and posterior cingulate cortex (*t* = 7.4 and 6.8), as shown in Figure 3A. Similar ROIs were found in the hippocampus, fusiform gyrus, middle temporal gyrus and left posterior cingulate cortex for another two contrasts of MCI-vs.-HE and AD-vs.-MCI, but with lower *t*-values. Group *t-statistics* of PiB-PET scans for AD-vs.-HE contrast suggested significant differences in the majority of cortical regions e.g., parietotemporal, frontal gyrus and posterior cingulate cortex (Figure 3B). As expected, similar ROIs were suggested by the results of the other two contrasts and with lowest *t-values* for AD-vs.-MCI. This is consistent with results from several previous works (Frisoni et al., 2010; Nordberg et al., 2010; Hinrichs et al., 2011; Bateman et al., 2012; Cohen and Klunk, 2014; Stam, 2014; Hafkemeijer et al., 2015), and provides confidence in our subsequent analyses.

### Multimodal MKL Analysis Enhanced Classification Accuracy

**Figure 4:**
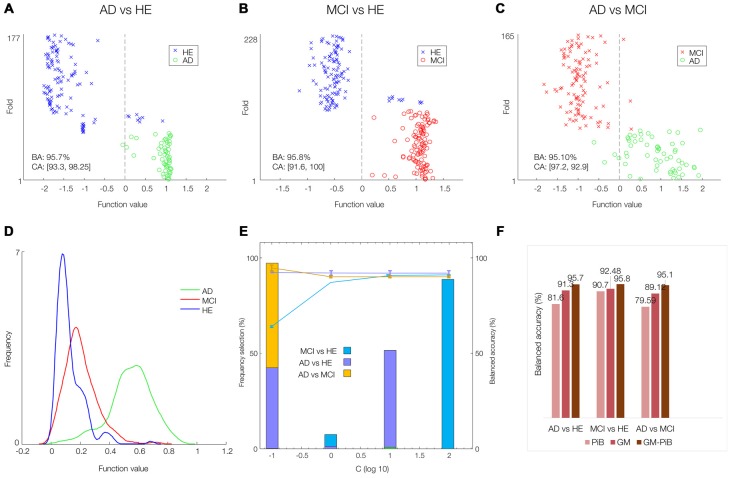
AD classification by simple multi-kernel learning (sKML) using combined GM-PiB imaging data. **(A–C)** Prediction plots (per fold). The decision threshold is displayed by a vertical line at the center of the plot. **(D)** Corresponding histograms of the function values of three groups modeled by sMKL. **(E)** Performance curve depending on the hyper-parameter values (*C* = 0.1, 1, 10, 100) with frequency of selection of each hyper-parameter, for three binary classification problems. **(F)** A summary of classification accuracies obtained by single and multi-modal data.

The sMKL was applied to multimodal GM-PiB of 58 AD, 108 MCI and 120 HE subjects. Figures 4A–D show scatter plots (prediction per fold) and their corresponding histograms of the function values obtained for the three contrasts. Note that scatter prediction plot in Figures 4A–C represent the predicted values (*x*-axis) against the real values or targets (*y-axis)* values, whereas histogram plots in Figure 4D are the smoothed density versions of the prediction plot that indicate how the function values were distributed. The performance curve with the frequency of selection of each hyper-parameter is shown in Figure 4E. In our data, margins with the SVM parameter *C* = 10^2^ resulted in a stable model performance across three classification problems. Promisingly, multi-modal analysis yielded the balanced accuracies (BAs) of around 95% for all three classification problems: 95.7% (CA_1_ = 93.3, CA_2_ = 98.2) for AD-vs.-HE, 95.81% (CA_1_ = 91.6, CA_2_ = 100) for MCI-vs.-HE, and 95.1% (CA_1_ = 97.2, CA_2_ = 92.9) for AD-vs.-MCI contrasts, which with respect to single-modal analysis modeled by SVM was considerably higher (SVM for PiB scans yielded BAs of 79.59% in AD-vs.-MCI, 90.07% in AD-vs.-HE, and 81.6% in MCI-vs.-HE, and SVM for GM scans resulted BAs of 89.12% in AD-vs.-MCI, 92.48% in AD-vs.-HE, and 91.3% in MCI-vs.-HE). For ease of comparison, the BAs obtained based on single- (GM or PiB) and multi-modal (GM + PiB) analysis are summarized in Figure 4F supporting the superiority of the multi-modal classification to single-modal analysis.

**Figure 5:**
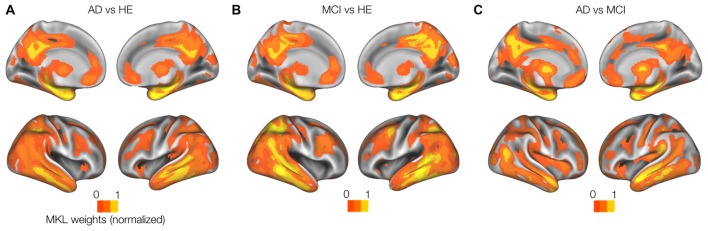
Weight (per region) maps modeled by multi-kernel learning (MKL) using GM-MRI data. Rendered MKL weights on a template. The results from a single modality GM-MRI data with an average of 75% contribution to **(A)** AD-vs.-HE, **(B)** MCI-vs.-HE and **(C)** AD-vs.-MCI classification problems. Weights for PiB-PET with lower (25% or less) contribution are not shown.

Whole-brain model weights obtained by sMKL (per region) from GM modality in the AD-vs.-HE contrast suggested ROIs at left hippocampus (1.85% ROI weight and 2221 voxels), right hippocampus (1.52%, 2296 voxels), left posterior cingulate cortex (1.61%, 4364 voxels), right posterior cingulate cortex (1.54%, 3557 voxels), right parahippocampus (1.43%, 2557 voxels), left parahippocampus (1.30%, 2344 voxels), left inferior occipital (1.22%, 2264 voxels), as in Figure 5A. In addition, sMKL weights from PiB-PET in the AD-vs.-MCI contrast suggested a bilateral temporal gyrus (left with 2.5% and 120 voxels and right with 2.05% and 132 voxels) and bilateral mid-frontal gyrus (left with 1.68%, 1206 voxels, right with 2.23%, 1100 voxels). The ROIs detected during MCI-vs.-HE and AD-vs.-MCI were analogous to that during AD-vs.-HE for each data modality, but with relatively lower weight values (Figures 5B,C). sMKL suggested a greater contribution by GM modality (a contribution level of 74.6 ± 3.1 (mean ± SD) and with an expected ranking of 0.9961 ± 0.3) than the PiB modality (a contribution level of 25.3 ± 4.9 and with an expected ranking of 1.99 ± 1.1) in classifying three groups of subjects.

### MKL Multi-Modal Analysis Improves Prediction Accuracy

**Figure 6:**
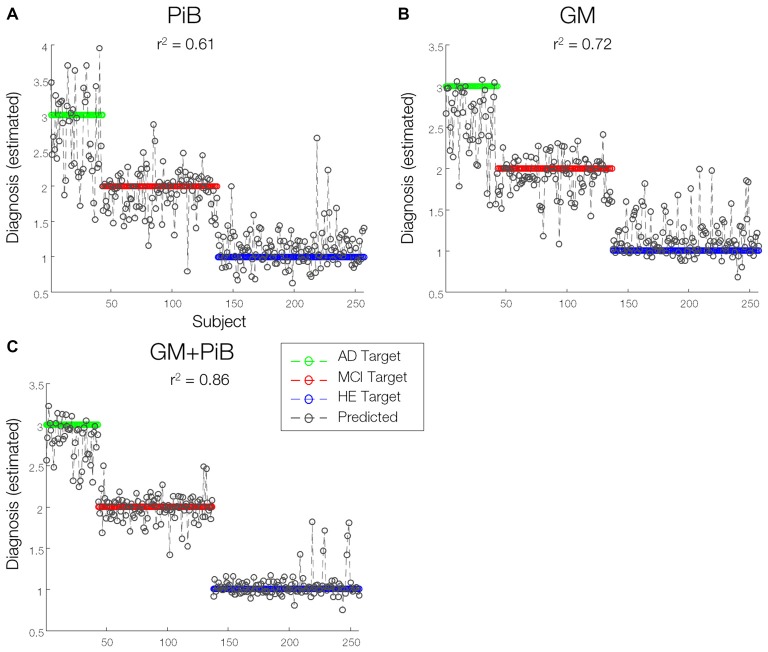
Predictions of diagnosis of individuals. A line prediction plot (predictions overlaid on targets) of diagnosis values of subjects derived from **(A)** GMs **(B)** PiB-PET scans modeled by kernel ridge regression (KRR) method and **(C)** combined GM-MRI and PiB-PET data modeled by sMKL. Proximity of sample data to any colored horizontal line denotes the likelihood of classifying under that particular diagnostic category associated with that line.

**Figure 7:**
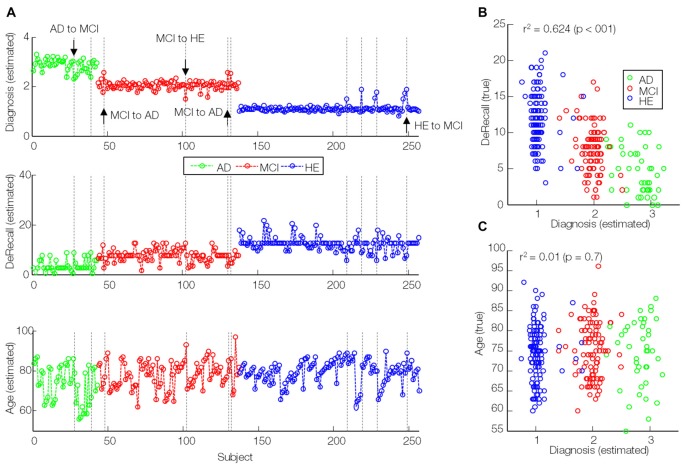
Correlation of estimated diagnosis values (modeled by sMKL) with true DeRecall scores and age values. **(A)** Line plots of estimated diagnosis values (similar to Figure 6C), **(B)** true DeRecall scores and **(C)** true age values. Dashed lines: outlier (transition candidates) samples. Down/upside arrows: correctly detected transitions.

The sMKL regression analysis applied to combined GM and PiB scans provided a correlation of *r*^2^ = 0.86 (*p* < 0.01) for the estimated diagnosis values, an improved prediction accuracy with respect to data based on individual participant (*r*^2^ = 0.72, *p* < 0.01 using GM and *r*^2^ = 0.61, *p* < 0.01 using PiB, modeled by KRR), as in Figure 6. Yet, we noticed some outliers in the predicted values or mispredicted samples (Figure 7A). Interestingly, 3/4 (2 MCI to AD and 1 MCI to HE) of MCI converters, 1/2 (AD to MCI) of AD converters and 1/4 (HE to MCI) of HE converters were correctly identified by the regression model. We hypothesized that the samples expected to be identified as HE but with predicted values closer to MCI had lower DeRecall scores than the average of the expected group i.e., HE. Conversely, the sample expected to be MCI but with estimated values closer to HE had cognitive scores relatively higher than the average of the MCI group. To test this hypothesis, we examined Pearson correlation between estimated values and their corresponding non-categorical variables, DeRecall and age values. We found a significant correlation between the estimated diagnostic values and the DeRecall scores (*r*^2^ = 0.62, *p* < 0.01), but no (significant) correlation with age values (*r*^2^ = 0.01, *p* = 0.7), as in Figures 7B,C.

**Figure 8:**
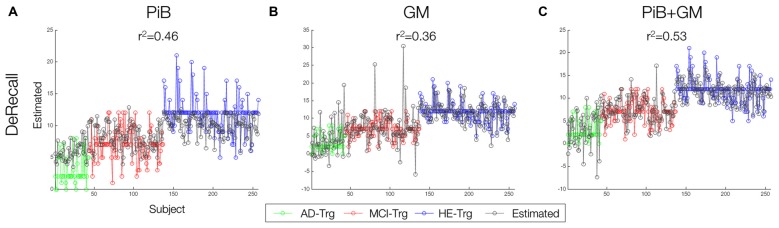
Predictions of three target values using single and multi-modal data. **(A–C)** Line prediction plot of delay memory recall scores. Individual GM an PiB modeled by KRR while multimodal GM-PiB modeled by sMKL. The closer a particular predicted data (gray) to the targeted data (in green, red and blue), the better the accuracy of sample data.

Finally, DeRecall scores were modeled by the sMKL regression for the combined GM-PiB data. Results were compared with a KRR regression model for individual modality data, GM and PiB (Figure 8). The multimodal analysis and sMKL provided the best regression accuracy for all three target values with *r*^2^ = 0.53 for DeRecall. This is compared to the lower values obtained from the individual modality data and KRR analysis with *r*^2^ = 0.46 and *r*^2^ = 0.36 for the DeRecall, using PiB and GM, respectively.

## Discussion

In the current work, we have applied a machine-learning framework based on multimodal analysis for AD classification and prediction of the AIBL data. Crucially, we utilized the Dartel algorithm to enhance coregistration of structural MRI and PET scans while the MKL technique combines complementary data information from different modalities to improve AD classification and prediction (Ye et al., 2008). In particular, we combined GM and PiB data from the AIBL dataset. At least for the AIBL data, our integrated Dartel-MKL multimodal approach revealed a very high classification accuracy (>95%) for the three binary diagnostic classification problems (Figure 4). Importantly, it could potentially predict the diagnosis of individuals, and their potential transition across diagnostic categories in the future (in 18 months or later), although more data are required to confirm this.

Although previous works have successfully tested the Dartel algorithm for AD classification in combination with MKL, there is no work that has tested on the AIBL data. Further, it was applied to only structural data (Cuingnet et al., 2011). Using voxel-based or cortical thickness features extracted from combined GM, WM and CSF, a classification rate of up to 81% sensitivity and 95% specificity was achieved for four groups of subjects including AD, MCI converters, MCI non-converters and HE. However, compared with GM alone, including all tissue maps provided slight improvement in the classification performance. In our work, we selected GM features as representative of the structural data, supported by the ANOVA test (Figure 2). Moreover, we included PET (PiB) scans to complement the analysis and improve the quality of diagnosis. In another work that used voxel-based features of GM and FDG data, an average classification accuracy of 94% was achieved for three groups of subjects including AD, frontotemporal lobar degeneration and control subjects (Dukart et al., 2011). However, the features used in their multimodal analysis were directly concatenated into a long feature vector and not formally combined. Importantly, the concatenation can increase the dimensionality of the feature space, making the classifier unstable and lead to overfitting. In contrast, MKL provides a unified way to combine heterogeneous data when different types of data cannot be directly concatenated. In addition, it provides more flexibility by using different weights on biomarkers of different modalities (Shen et al., 2014).

Our whole-brain investigations on group statistical differences using GMs and PiB scans (Figure 3) suggested MRI and PET changes in the regions that are known to be affected by AD e.g., temporal gyrus, (para)hippocampus, lingual gyrus, thalamus, posterior cingulate cortex and amygdala, consistent with previous findings (Frisoni et al., 2010; Nordberg et al., 2010; Hinrichs et al., 2011; Bateman et al., 2012; Cohen and Klunk, 2014; Stam, 2014; Hafkemeijer et al., 2015). Our MKL and multimodal analysis provided a high regression accuracy (*r*^2^ = 0.86) for AD classification (Figures 6, 7), as a rough measure of multi-class separation. We found high negative correlations between the estimated values and a well-known cognitive test score, DeRecall, with *r*^2^ = 0.62 (Figure 7B). However, we found no significant correlation (*r*^2^ = 0.02) between the estimated diagnostic category and age (Figure 7C). Importantly, those correlations can justify the sample outliers provided by the regression model. For example, samples that were expected to be identified as HE but estimated with values close to MCI, their DeRecall score were relatively lower than the average of the target group. Conversely, samples expected to be MCI but with estimated values closer to HE their cognitive scores were relatively higher than the average of the expected group values i.e., MCI (Figure 7A). These findings suggest that the proposed framework could potentially help in predicting individual baseline diagnoses and likelihood of stability or conversion. Future work will test this on a larger dataset or on follow-up analysis.

Another multimodal classification method, different from the MKL method, called multimodal support vector classification (SVC) had been introduced as a simple and effective way of combining various data sources (Zhang et al., 2011). Feature selection (not optimization) was performed under a coarse-grid searching via cross validation. Tested for combined GM, FDG and CSF biomarkers from the ADNI data, a high classification accuracy (93.2%) for AD-vs.-HE but a relatively low accuracy (76.4%) for MCI-vs.-HE subjects were achieved. In this work, the features were simply the volume of GM tissue and average intensity of FDG scans in 93 ROIs added with features from CSF biomarkers, i.e., a total of 93 + 93 + 3 features for each participant. In general, region-based features were more intuitive and suitable for *post hoc* analysis. However, cross similarities of the samples were indirectly generated by a (linear or Gaussian) kernel function. In comparison, the values of the similarity matrix were coarser, representing whole-brain activities, but directly account for the interactions among samples. Nevertheless, for a better evaluation, future work should compare the performance of two SVC and MKL methods under two region-based and whole-brain strategies, using a similar dataset e.g., ADNI or AIBL.

An extension of SVC called support vector regression (SVR) under a multi-modal multi-task learning (M3T) was introduced (Zhang and Shen, 2012). It was tested on the ADNI dataset (similar to Zhang et al., 2011) and provided improved correlations between biomarkers from multimodal data (GM + FDG + CSF) and MMSE (*r* = 0.69) and AD assessment scale-cognitive subscale (ADAS-Cog) score (*r* = 0.73), with respect to individual data. In comparison, our sMKL method applied to GM + PiB provided higher correlations between kernels and psychological test scores (*r*^2^ = 0.62 for DeRecall). Again, for a better evaluation, future works should compare the two methods under similar conditions and over a similar dataset. Another multimodal approach for AD classification is a classical multidimensional scaling (to generate joint embedding) and random forest-based algorithm (for classification), which was tested on four different modalities including MRI, FDG-PET, CSF biomarkers and categorical genetic information in the ADNI data (Gray et al., 2013). It would perhaps be worthwhile to more closely compare this method with the MKL and multi-kernel SVM methods.

In our study, PiB-PET scans were masked with a whole brain template where only non-informative features from outside the brain were discarded. However, it is known that brain tissues (GM, WM and CSF) can have different uptake patterns (Klunk et al., 2004). Hence this is clearly a limitation of our method. As a solution, an additional normalization, standardized uptake value ratio (SUVR) has been suggested (Raniga et al., 2008; Dore et al., 2012). Under PiB-SUVR normalization, uptake values are normalized by the mean uptake value within a region containing nonspecific binding e.g., cerebellar gray matter (Perani et al., 2014). This leads to better inter-subject variability, suitable for group analysis. However, this normalization requires a careful (tissue) segmentation and brain parcellation. In future work, this will be incorporated as an extension to our current framework to improve performance of the multimodal analysis.

Our study can also be extended in two aspects. First, due to a high level of accuracy offered by our multimodal analysis, subtle changes (mild symptoms) that are evident at an early stage of the disease can be investigated (Davatzikos et al., 2011). This may eventually improve the diagnostic quality. Second, as significant correlation was found between imaging measures with known psychological test scores such as DeRecall, incorporating such features into the machine-learning framework (Figure 1) may potentially further improve the diagnosis. This can be achieved as a contributing (weight) factor for the decision function of the (SVM) classifier or as an independent data modality, similar to the CSF biomarkers in Zhang et al. (2011) to enhance the multimodal analysis.

To conclude, our work has shown that a proper combination of Dartel and sMKL methods applied to the multimodal neuroimaging data in AIBL can substantially improve the classification of AD of not only group samples but also individual samples. There is also a potential application for predicting individual conversions. Hence, this could likely lead to direct clinical applications, for example, in the form of a clinical decision support tool. Our work provides further support in favor of machine learning, particularly MKL-based, approach in improving the diagnosis and risk prediction of AD. The computational approach is also sufficiently general to allow incorporating other e.g., non-imaging data features to further improve the performance.

## Software Note

Part of the implementation (preprocessing, DARTEL and MKL analyses) have been unified under a publicly available MATLAB graphical user interface, named Software Integrating NEuroimaging And other Data (SINEAD) accessible at github.com/vyoussofzadeh/SINEAD_tool.

## Author Contributions

VY conceptualized the methodological framework and carried out the bulk of analyses for the current study. He prepared the initial manuscript and carried out revisions after consulting with coauthors. BM and LPM advised on analyses, choice of metrics and provided edits on the manuscripts during development. KFW-L led the project and oversaw all aspects of the study, from conceptualization, data analysis, writing and editing this manuscript.

## Conflict of Interest Statement

The authors declare that the research was conducted in the absence of any commercial or financial relationships that could be construed as a potential conflict of interest.

## Footnotes

^1^www.fil.ion.ucl.ac.uk/spm/
