# Corrigendum: Multi-Kernel Learning with Dartel Improves Combined MRI-PET Classification of Alzheimer's Disease in AIBL Data: Group and Individual Analyses

**DOI:** 10.3389/fnhum.2017.00457

**Published:** 2017-09-08

**Authors:** Vahab Youssofzadeh, Bernadette McGuinness, Liam P. Maguire, KongFatt Wong-Lin

**Affiliations:** ^1^Computational Neuroscience Research Team, Intelligent Systems Research Centre, School of Computing and Intelligent Systems, Faculty of Computing and Engineering, Ulster University Londonderry, United Kingdom; ^2^Division of Neurology, Cincinnati Childrens Hospital Medical Center Cincinnati, OH, United States; ^3^Institute of Clinical Science B, Centre for Public Health, Queens University Belfast Belfast, United Kingdom

**Keywords:** Alzheimer's disease, classification, machine learning, multi-kernel learning, prediction, Australian imaging, biomarkers, lifestyle AIBL

There are incorrect details in the Acknowledgments Section. The first sentence should read:

This work was performed under the Northern Ireland International Health Analytics Centre (IHAC) collaborative network project funded by Invest NI through Northern Ireland Science Park (Catalyst Inc.).

Wherein “New York, NY, USA” has been removed from the sentence.

The original article has been updated.

## Conflict of interest statement

The authors declare that the research was conducted in the absence of any commercial or financial relationships that could be construed as a potential conflict of interest.

